# The association between gait speed and falls in ambulatory adults with spinal muscular atrophy: a retrospective pilot study

**DOI:** 10.3389/fneur.2024.1491466

**Published:** 2024-12-04

**Authors:** Kathryn Jira, Andrea Jaworek, Matti Allen, Songzhu Zhao, Kristina Kelly, W. David Arnold, Bakri Elsheikh

**Affiliations:** ^1^Department of Neurology, The Ohio State University Wexner Medical Center, Columbus, OH, United States; ^2^Center for Biostatistics, The Ohio State University Wexner Medical Center, Columbus, OH, United States; ^3^NextGen Precision Health, University of Missouri, Columbia, MO, United States

**Keywords:** spinal muscular atrophy, falls, fatigability, neuromuscular, gait, ambulation, 6-Minute Walk Test

## Abstract

**Introduction:**

Fatigue and gait speed are established determinants of fall risk in patients with neurological disorders. However, data on adults with spinal muscular atrophy (SMA) is limited. The aim of this pilot study was to investigate falls and risk factors in adults with SMA.

**Methods:**

A retrospective chart review of ambulatory adults with genetically confirmed 5q-SMA included: age, sex, age of symptom onset, SMN2 copy number, BMI, and 6MWT distance and speed at minutes 1, 2, and 6.

**Results:**

Fourteen ambulatory patients were included in the analysis with an average follow-up of 36 months (range of 12–66 months). 10 patients were classified as fallers (F_all_) and four as non-fallers (NF_all_). One faller received Risdiplam, while the remaining fallers and non-fallers received Nusinersen for the duration of the follow-up period. In the F_all_ cohort, the median speed at 1, 2, and 6 min were 0.92 m/s, 0.89 m/s, and 0.77 m/s, respectively, with a heterogeneous range including one faller at 1.25 m/s. In the NF_all_ cohort, the slowest collected median recorded speeds were 1.18 m/s, 1.11 m/s, and 1.09 m/s respectively, with one non-faller at 0.56 m/s. There was no significant statistical difference between 6-min gait speeds and individuals experiencing falls. However, we found a three-fold greater decline in speed between the 6MWT first and last minute in the F_all_ (13.01%) compared to the NF_all_ (5.16%). 7 of 10 patients had multiple falls (70%) with two individuals consequently losing ambulation (20%).

**Discussion:**

These findings underscore the need for larger studies on fatigability and the importance of considering factors beyond gait speed alone.

## 1 Introduction

Spinal muscular atrophy (SMA) is an autosomal-recessive degenerative neuromuscular disease affecting lower motor neurons due to the absence of the survival of motor neurons (SMN1) gene (1). This results in progressive denervation and muscle weakness, predominately affecting the proximal muscles (2). SMA occurs in 1 out of 10,000 to 20,000 live births (3, 4), with age of onset and clinical severity often correlated with the number of survival motor neurons 2 (SMN2) copies (5).

Those individuals with one or two SMN2 copies are typically non-ambulatory, whereas individuals with three to four SMN2 copies can walk either household or community distances (6). Although this trend is noted, recent systematic review suggests that SMN2 copy number alone does not predict walking ability (7). Studies have also found that sex, along with SMN2 copy number, is a predictor of disease severity related to ambulatory function, with males more severely affected than females (5, 8). Although it is not clear how sex-based differences are affected by ambulatory function, specifically.

As the disease progresses, individuals with SMA may lose their ability to ambulate due to weakness, fatigue, or injuries resulting from falls. So far, clinicians have not been able to accurately predict loss of ambulation in SMA. A history of falls is often reported prior to complete loss of ambulation (9, 10). Limited research assessed the association between ambulation and falls in SMA, including a multiple-case study design of five adults and two children showing that strength, gait velocity, and variability in gait pattern were not predictors of falls but stride-length variability was a significant risk factor (9). In contrast, gait speed is a validated predictor of fall risk in other neurological diseases such as Huntington’s disease (11, 12), Parkinson’s disease (13, 14), and stroke (15).

Within neuromuscular disease, there is limited data on gait speed in adults. One study assessed children with Charcot–Marie Tooth Disease finding a gait speed of 0.94 m/s, significantly slower when compared to healthy controls (16). Another study found that gait speed was slower and stride length was shorter in children with neuromuscular disease (Duchenne Muscular Dystrophy, Becker Muscular Dystrophy, and Charcot–Marie-Tooth) compared to typical developing peers (17). In healthy adults between 30 and 39 years old, gait speeds tend to range from 1.32 to 1.55 m/s in men and 1.19 to 1.48 m/s in women (18). Clinicians use the 10 Meter Walk Test or 6 Minute Walk Test (6MWT) to assess gait speed and stratify an individual as a community or household ambulatory. In healthy adults, those with a gait speed of <0.8 m/s were found to be at an increased risk of falling and were classified as household ambulators (19). In an upper and lower motor neuron disease such as amyotrophic lateral sclerosis (ALS), changes in gait including shorter stride length, increased stride duration, slower walking speed, and increased gait variability were noted in comparison to healthy adults (20–23). These spatial–temporal changes in gait were associated with lack of independence, lower quality of life, and increased risk for falls (24). A study investigating the changes in lower extremity function across clinical phenotypes in ALS also found that a history of falls serves as an early indicator of disease progression and severity (25). Assessing fall risk in the neuromuscular population is important as it can signal a progressive loss in ambulation (25).

Fatigue, defined as an overwhelming sense of tiredness, lack of energy, feeling of physical or mental exhaustion or both (26–28), is reported in 81–100% of patients with SMA and found to be a major barrier for participation in physical activities, recreational activities, and overall mobility (29–31). The term fatigue can be broken down into two domains: physical and mental fatigue (26). Physical fatigue can be captured as fatigability which can be measured by the performance on an outcome measure (26). Studies have used the 6MWT to capture rate of speed decline across the minutes of the test as a means of capturing fatigability in ambulant patients. One study found a 17% decline in speed between the first minute and the last minute of the 6MWT in individuals with SMA, capturing fatigability during ambulation (32). In a healthy cohort, a study found a typical percent decline in speed from the first minute to the last minute during the 6MWT was 1.4% (33).

However, to date, no study has been able to associate fall risk with a quantifiable value in SMA. The purpose of this study was to investigate falls in adults with genetically confirmed 5q-SMA and the predicting factors associated with falls. We hypothesized that individuals with reported falls would express greater fatigability and slower gait speed when compared to individuals who are not falling.

## 2 Materials and methods

### 2.1 Study design

A retrospective chart review of 53 charts was conducted at the Ohio State University Wexner Medical Center. This study was approved by the institutional review board. The study period occurred between 2017 and 2023.

### 2.2 Study population

Inclusion criteria included: age > 18 years old, genetic confirmation of 5q-SMA, and ability to ambulate at least 10 meters with or without an assistive device. Exclusion criteria included: lack of follow-up post baseline assessment.

### 2.3 Study overview

Collected data included age, sex, initial age at symptom onset, SMN2 copy number, body mass index (BMI), and 6MWT (34, 35) distance in individuals that report falls or no falls with an average follow up of 36 months (range of 12–66 months). Clinic visits occurred on average every 6 months post baseline visit. During the clinic visits, the functional assessments were performed according to standardized practice by neurologic physical therapists who specialize in neuromuscular conditions (35). A fall history was obtained by the physical therapist or physician asking the patient if they had experienced any falls since their last visit and if any of those falls led to injuries or hospitalizations. For the 6MWT, individuals were permitted to use an assistive device if the individual used one daily. To assess gait speed and fatigability, the 6MWT speeds at 1, 2, and 6 min were used for analysis during the visit preceding the reported fall to best predict the speed at which the individual was ambulating with prior to their fall. Multiple time points with the 6MWT were analyzed to attempt to capture the possible fatigability by assessing the change in gait speed over time. Fatigability was represented as the percent difference in speeds between minutes 1 and 2, minutes 2 and 6, and minutes 1 and 6. We recorded the pre-fall gait speed as it best reflects their gait characteristics preceding the fall. We recorded the slowest speed to investigate a minimum gait speed that does not correspond with fall. We also recorded the fastest speed to document a range in ambulation. Additionally, we calculated the median gait speed for each individual as a robust measure of central tendency. As a comparison, everyone’s slowest, fastest and median 6MWT speeds at 1, 2, and 6 min were analyzed in both cohorts. Standardized motivation was provided at each minute of the test.

### 2.4 Statistical analysis

A chi–squared test was used to compare categorical variables. For continuous variables, 2-sampled *t*-tests (for normally distributed data) and the Mann – Whitney *U* test (for non-normally distributed data) were used to compare the faller and non-faller groups.

## 3 Results

Fourteen ambulatory patients (9 men, 5 women; median age 33.50; range 17–48) with SMA met enrollment criteria and were classified as fallers (F_all_, *n* = 10) and non-fallers (NF_all_, *n* = 4) based on subjective report during clinic visits. Patients had clinic visits every 6 months for a minimum of 2 years. The average follow-up time was 36 months (range 12–66 months). All participants were receiving Nusinersen except for one in the fall cohort who was receiving Risdiplam consistently throughout the entire study period. None of the participants used assistive devices during the 6MWT. However, in the community for longer distances, 60% of F_all_ cohort used wheelchairs compared to 25% in the NF_all_ cohort. Age, sex, initial age at symptom onset, and BMI were similar between the falling and non-falling cohorts as shown in Table 1. All patients with 3 SMN2 copies were in the fall cohort, whereas people with 4 or more copies were distributed between the two cohorts.

**Table 1:** Comparison of baseline demographics and gait speeds between the cohorts.

Variable	Level	Non-fallers (*n* = 4)	Fallers (*n* = 10)	Total (*n* = 14)	Kruskal-Wallis *p*-value
Baseline demographics					
Age	Median [IQR] (min, max)	missing = 0 37 [24.5, 45] (17, 48)	missing = 0 33.5 [24, 36] (21, 48)	missing = 0 33.5 [24, 42] (17, 48)	0.8315
Sex	F	1 (25%)	4 (40%)	5 (35.71%)	
	M	3 (75%)	6 (60%)	9 (64.29%)	
Initial age of onset	Median [IQR] (min, max)	missing = 0 4.5 [3.5, 11] (3, 17)	missing = 0 5.5 [3, 9] (2, 28)	missing = 0 5 [3, 9] (2, 28)	1.0000
SMN2	Missing	0 (0%)	1 (10%)	1 (7.14%)	
	3	0 (0%)	3 (30%)	3 (21.43%)	
	4	4 (100%)	5 (50%)	9 (64.29%)	
	5	0 (0%)	1 (10%)	1 (7.14%)	
BMI	Median [IQR] (min, max)	missing = 0 25.63 [23.22, 28.12] (23.06, 28.36)	missing = 1 25.11 [23.22, 40.1] (19.05, 40.75)	missing = 1 25.11 [23.22, 31.4] (19.05, 40.75)	0.6434
Comparison of gait speeds among cohorts					
*speed_1min (m/s)	Median [IQR] (min, max)	Missing = 1 1.18 [0.77, 1.4] (0.77, 1.4)	Missing = 0 0.92 [0.66, 1.25] (0.42, 1.35)	Missing = 1 0.98 [0.77, 1.25] (0.42, 1.4)	0.3967
*speed_2min (m/s)	Median [IQR] (min, max)	Missing = 0 1.11 [0.67, 1.27] (0.28, 1.38)	Missing = 0 0.89 [0.52, 1.2] (0.34, 1.34)	Missing = 0 1.01 [0.52, 1.2] (0.28, 1.38)	0.6714
*speed_6min (m/s)	Median [IQR] (min, max)	Missing = 0 1.09 [0.82, 1.25] (0.56, 1.38)	Missing = 0 0.77 [0.36, 1.11] (0.22, 1.32)	Missing = 0 0.99 [0.49, 1.12] (0.22, 1.38)	0.2579
*change_1_6	Median [IQR] (min, max)	Missing = 1 5.16 [1.79, 26.45] (1.79, 26.45)	Missing = 0 13.01 [2.51, 33.33] (0.67, 66.37)	Missing = 1 11.36 [2.51, 27.88] (0.67, 66.37)	0.4990
*change_2_6	Median [IQR] (min, max)	Missing = 0 2.1 [0.2, 51.41] (0, 99.02)	Missing = 0 11.17 [1.42, 25] (0.35, 57.37)	Missing = 0 5.74 [0.67, 25] (0, 99.02)	0.4795
*change_1_2	Median [IQR] (min, max)	Missing = 1 1.79 [1.41, 63.04] (1.41, 63.04)	Missing = 0 2.98 [0.59, 12.24] (0, 21.12)	Missing = 1 2.12 [1.11, 12.24] (0, 63.04)	0.6116
Fastest speed 1 min	Median [IQR] (min, max)	Missing = 0 1.39 [1.04, 1.54] (0.81, 1.57)	Missing = 0 0.93 [0.8, 1.25] (0.45, 1.49)	Missing = 0 1.09 [0.81, 1.27] (0.45, 1.57)	0.1194
Fastest speed 2 min	Median [IQR] (min, max)	Missing = 0 1.37 [1, 1.52] (0.76, 1.55)	Missing = 0 1 [0.73, 1.25] (0.42, 1.46)	Missing = 0 1.15 [0.76, 1.27] (0.42, 1.55)	0.1371
Fastest speed 6 min	Median [IQR] (min, max)	Missing = 0 1.31 [0.98, 1.46] (0.72, 1.54)	Missing = 0 0.95 [0.47, 1.26] (0.39, 1.4)	Missing = 0 1.08 [0.63, 1.26] (0.39, 1.54)	0.1573
Slowest speed 1 min	Median [IQR] (min, max)	Missing = 1 1.18 [0.77, 1.4] (0.77, 1.4)	Missing = 0 0.8 [0.55, 1.05] (0, 1.35)	Missing = 1 0.87 [0.59, 1.14] (0, 1.4)	0.1282
Slowest speed 1 min	Median [IQR] (min, max)	Missing = 1 1.18 [0.77, 1.4] (0.77, 1.4)	Missing = 0 0.8 [0.55, 1.05] (0, 1.35)	Missing = 1 0.87 [0.59, 1.14] (0, 1.4)	0.1282
Slowest speed 2 min	Median [IQR] (min, max)	Missing = 0 1.11 [0.67, 1.27] (0.28, 1.38)	Missing = 1 0.84 [0.52, 1.04] (0.35, 1.34)	Missing = 1 0.94 [0.52, 1.14] (0.28, 1.38)	0.3545
Slowest speed 6	Median [IQR] (min, max)	Missing = 0 1.09 [0.82, 1.25] (0.56, 1.38)	Missing = 0 0.55 [0.3, 1] (0.13, 1.32)	Missing = 0 0.69 [0.35, 1.12] (0.13, 1.38)	0.1198
Median speed 1 min	Median [IQR] (min, max)	Missing = 0 1.33 [1.01, 1.45] (0.78, 1.49)	Missing = 0 0.92 [0.72, 1.14] (0, 1.42)	Missing = 0 1.04 [0.78, 1.25] (0, 1.49)	0.1198
Median speed 2 min	Median [IQR] (min, max)	Missing = 0 1.28 [0.93, 1.42] (0.7, 1.46)	Missing = 0 0.92 [0.62, 1.12] (0.42, 1.4)	Missing = 0 1.05 [0.7, 1.17] (0.42, 1.46)	0.1194
Median speed 6 min	Median [IQR] (min, max)	Missing = 0 1.25 [0.89, 1.39] (0.62, 1.44)	Missing = 0 0.85 [0.36, 1.12] (0.14, 1.36)	Missing = 0 1 [0.56, 1.18] (0.14, 1.44)	0.1198

*Slowest for patient without falls and the last assessment before the reported fall for those with falls.

No falls or injuries occurred during the testing. The distances walked and corresponding gait speeds at 6 min between the falling and non-falling groups were not associated with predicting fall risk as shown in Table 1.

In the F_all_ cohort, 7 of the 10 patients (70%) had multiple falls over the course of the study period, with two (20%) of those individuals having falls sustaining serious lower extremity fractures resulting in hospitalizations and loss of ambulation. Per physical therapy and physician documentation, no other serious injuries or hospitalizations occurred in the remaining patients.

**Figure 1:**
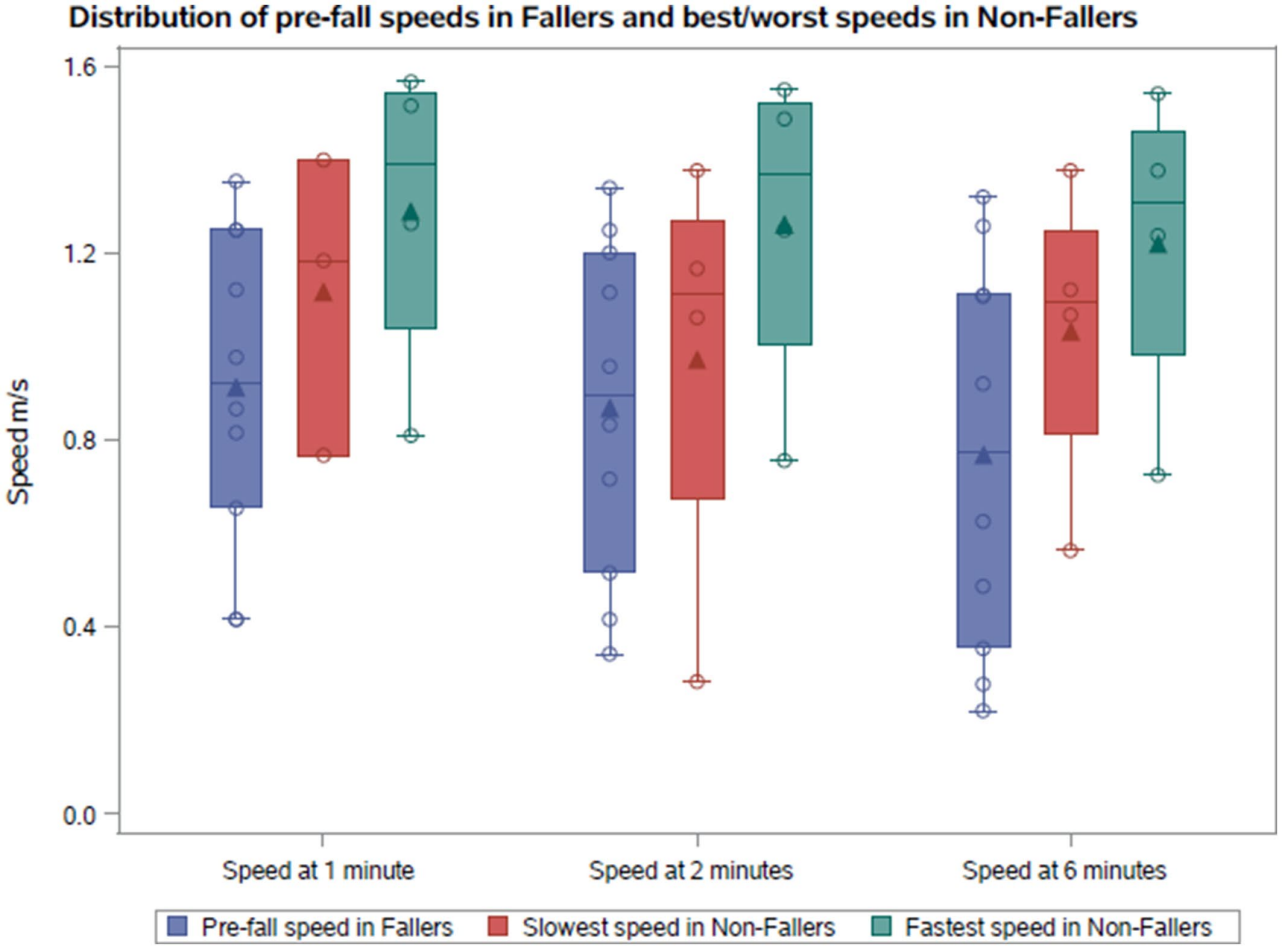
Comparison of the pre-fall gait speeds with the fastest and slowest gait speeds in non-fallers.

**Figure 2:**
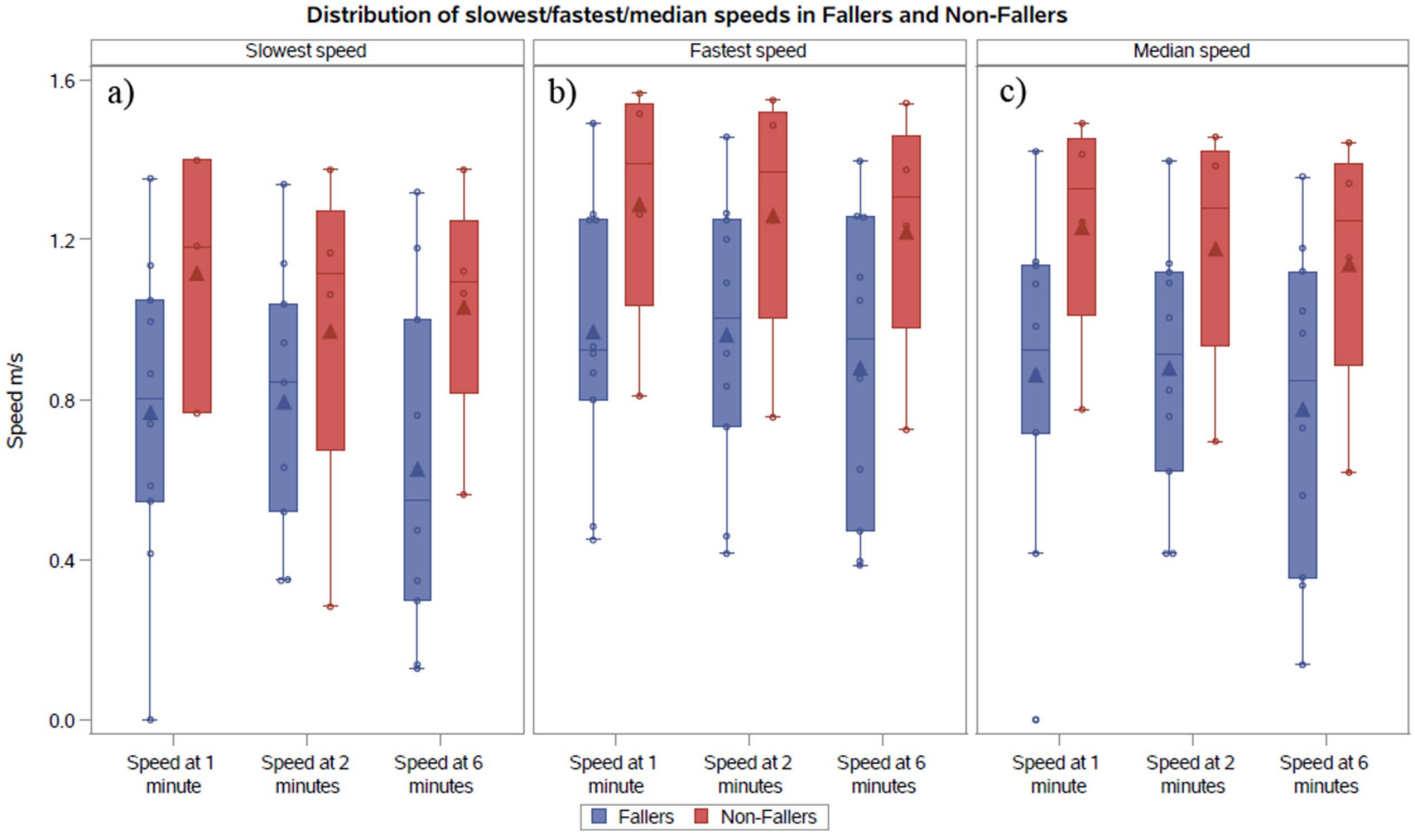
**(A)** Comparison of the slowest recorded gait speeds between the NF_all_ and F_all_ cohorts. **(B)** Comparison of the fastest recorded gait speeds between the NF_all_ and F_all_ cohorts. **(C)** Comparison of the median gait speeds between the NF_all_ and F_all_ cohorts.

The gait speeds across minutes 1, 2, and 6 in the 6MWT are illustrated in Figures 1, 2 for both cohorts. We compared the median pre-fall gait speed in the F_all_ cohort to the slow and fast median gait speeds of the NF_all_ cohort to decern if there was a range of speeds where patients are at a higher risk for falls. Despite considerable overlap between pre-fall gait speeds for fallers and median speeds for non-fallers, a distinct trend emerged. In the F_all_ cohort, the median speeds at 1, 2, and 6 min, measured at the visit preceding the fall, were 0.92 m/s, 0.89 m/s, and 0.77 m/s, respectively, with one individual reaching 1.32 m/s at 6 min. For non-fallers, the median slowest speeds were 1.18 m/s, 1.11 m/s, and 1.09 m/s, and the median fastest speeds were 1.39 m/s, 1.37 m/s, and 1.31 m/s at minutes 1, 2, and 6, respectively. Therefore, we found that the slowest median speeds at 1, 2, and 6 min for non-fallers were nominally faster than the speed recorded prior to a fall. To further explore the distribution of gait speeds at 1, 2, and 6 min between fallers and non-fallers, the median gait speeds for each participant were calculated across study period and compared between the two groups. In the F_all_ cohort, the median speeds at 1, 2, and 6 min were 0.92 m/s, 0.92 m/s, and 0.85 m/s, respectively. For the NF_all_ cohort, the median gait speeds across the study period were 1.33 m/s, 1.28 m/s, and 1.25 m/s at minutes 1, 2, and 6, respectively. We therefore noted a trend where individuals who reported falls tended to have gait speeds below 1.0 m/s while those without reported falls tended to have gait speeds above 1.0 m/s shown in Table 1 and Figure 2C.

Similarly, a trend was observed where fallers walk with a slower gait speed than non-fallers. The slowest median speeds at 1, 2, and 6 min were 0.80 m/s, 0.84 m/s, and 0.55 m/s for fallers, compared to 1.18 m/s, 1.11 m/s, and 1.09 m/s for non-fallers as shown in Figure 2A. Whereas the fastest median gait speeds at 1, 2, and 6 min were 0.93 m/s, 1.0 m/s, and 0.95 m/s for fallers, compared to 1.39 m/s, 1.37 m/s, and 1.31 m/s for non-fallers shown in Figure 2B.

Additionally, we captured the percent difference in gait speeds across minutes 1, 2, and 6 during the 6MWT in both groups. Patients who fell showed a nominally greater decline in gait speeds between minutes 1 and 6, as well as 2 and 6, compared to non-fallers during the same intervals, though this difference was not statistically significant. One patient who reported falls demonstrated a 66.37% decline in gait speed from minute 1 to minute 6 in the 6MWT, which is substantially greater than the greatest percent decline in the NF_all_ cohort of 26.45%.

## 4 Discussion

Our retrospective study assessed the association between reported falls and both gait speed and fatigability in adults with SMA. We cannot draw firm conclusions due to the small sample size of 14 individuals. However, our findings highlight important trends found in the SMA population in regard to describing falls. There was not a statistically significant association between falls and gait speed in this small pilot study; however, we observed a tendency for NF_all_ cohort to have faster gait speeds compared to F_all_ cohort. In addition, we found that the F_all_ cohort had a greater relative decline in gait speed between the first and the last minute during the 6MWT compared to the NF_all_ cohort. In our study population, the majority of the F_all_ cohort experienced multiple falls, with some serious injuries resulting in loss of ambulation. Individuals who reported falls were more likely to own and use a wheelchair for community distances than individuals who did not report falls. This may indicate an increased reliance on assistive devices for mobility in those with a history of falls to reduce risk of injury. Further investigation should focus on analyzing gait and fatigue to assess for changes in ambulation that may require assistive devices or intervention to ensure safe ambulation.

### 4.1 Gait speed and fall risk

In various neurological conditions, there has been a well-established association between gait speed and fall risk. In diagnoses such as Huntington’s disease (11, 12), Parkinson’s disease (13, 14), and stroke (15), a gait speed under 1.0 m/s correlated with having a higher risk for falls. Our data showed similar trends. The majority of the patients in our F_all_ cohort had gait speeds less than 1.0 m/s, while all but one individual in the NF_all_ cohort had gait speeds >1.0 m/s as shown in Figure 2C. A larger sample size would allow for further exploration of this finding.

This observed trend highlights the importance of continuing to monitor gait speed over time to assess a patient’s decline in mobility. Our findings align with other publications in the neuromuscular disease population, which also report slower gait speeds compared to healthy controls (16, 17).

We noted that one individual in our F_all_ cohort reported multiple falls but demonstrated gait speeds >1.0 m/s. This suggests that working past the point of fatigability may contribute to fall risk. On the other hand, one individual reported no falls but demonstrated gait speeds <1.0 m/s, which might be related to higher fear of falling, leading to extra caution with walking. This raises the question should we be assessing fear of falling and other psychosocial factors to assess risk of falls? Falls are often a multifactorial event as a result of weakness, balance impairment, unforeseen perturbation, environmental context, perception of fatigue, fatigability, cognitive awareness, or equipment malfunction. Therefore, though a useful measure to identify a trend, gait speed alone should not be used as a sole predictor of fall risk. Future research should investigate other variables regarding psychometric and fall history measures as well as gait components. To assess for overall risk of falls, collecting a falls history, fear of falling assessment such as Falls Efficacy Scale-International, a balance assessment such as the Activity-Specific Balance Confidence Scale, and a general strength assessment would be recommended.

There are also multiple ways to assess gait speed. During the 6MWT, gait speed is approximated and averaged over time by dividing the total distance traveled by 360 s. Other studies have begun using wearable devices to capture real time information regarding stride length, step length, medial and lateral sway, tibial advancement, and gait speed. These devices have been proven to be more accurate and a reliable means of assessing gait, but researchers can be limited by cost and accessibility of the device. Future research should consider using wearable devices with a larger population to further investigate the association between gait speed and other gait parameters with predicting fall risk.

### 4.2 Fatigability, gait variability, and falls

This study attempted to capture fatigability by assessing the fatigability or change in speeds between the minutes of the 6MWT. Both the NF_all_ and F_all_ cohorts demonstrated a notable decline in gait speed between minutes 1 and 2, 1 and 6, and 2 and 6. Healthy adults were found to have a natural decline in gait speed by 1.4% between minutes 1 and 6 (28). In contrast, our study found that those who fall had a 13.01% decline in gait speeds between minutes 1 and 6, which is almost two and a half times greater than those who did not fall (5.16%). These findings suggest that both cohorts, regardless of fall history, have greater fatigability than healthy controls. A previous study found a 17% decline in speed between the first minute and the last minute of the 6MWT in individuals with SMA (9). However, that study did not separate individuals who did and did not report falls, making our finding relevant as they highlight the possible effect of fatigability on fall risk.

In our study, one of the individuals in the F_all_ cohort demonstrated a 66.37% decline in gait speed between minutes 1 and 6 of the 6MWT, while the greatest change in gait speeds between the same time points in the NF_all_ cohort was 26.45%. This highlights that fatigability may be a contributing factor in the decline in gait speeds. This aligns with Montes et al. in 2011 finding that fatigability shortens stride length resulting in slower gait speed (27). In addition, 7 of 10 patients in the F_all_ cohort reported multiple falls between clinic visits.

These findings also reflect greater gait variability among fallers compared to non-fallers. In a study assessing gait in ALS using wearable devices, patients displayed significantly higher gait variability specifically in swing and stance time compared to healthy controls (36). This study also investigated the affects of mild cognitive impairment on gait variability in single-and dual-task conditions (36). As expected, the magnitude of variability was greater during the dual-task condition compared to single-task (36). However, this study suggests that cognitive impairment effects gait variability and is independently associated with fall risk (36). In our study of SMA in a single-task environment, the change in gait speeds across the timed test represent gait variability. Participants in the F_all_ cohort expressed greater percent change in speed between minutes 1 and 6 (0.67–66.37%) when compared to the NF_all_ cohort (1.79–26.45%) in Table 1. Future studies should assess gait variability in SMA by capturing not only gait speed but also stride length, stability index, cadence, stance time, and swing time with wearable devices. Additionally, performing a gait analysis in the context of a dual-task environment may better predict fall risk in individuals with SMA as it may more accurately depict a community environment.

Future research should continue to use objective data, such as changes in gait speeds during 6MWT, with a larger population to identify a possible standard percent decline that would classify individuals as being at high risk of falls. In addition to objective measures, future research should also consider obtaining perceptions of fatigue such as using the Ratings of Perceived Exertion (RPE) scale, NeuroQol Fatigue Computer Adaptive Test (CAT), or Fatigue Impact Scale (FIS). Since fatigue contains both mental and physical components, utilizing subjective and objective measures would more accurately capture holistic fatigue.

### 4.3 Limitations

Due to the retrospective nature of the study, we were not able to capture the exact number of falls each participant experienced as we relied on the documentation from the physician and physical therapy notes. The small sample size impacted the power and significance of our statistical analysis. Also, we did not account for the potential influence of the different therapies, such as Nusinersen or Risdiplam, which could impact ambulation, though 13 out of 14 were on Nusinersen. When assessing fatigability, this study only considered changes in gait speed over time and did not account for subjective fatigue or muscle strength. Despite these limitations, the noted trends warrant further investigation to provide more meaningful conclusions in respect to guiding clinical practice and predicting fall risk.

## 5 Conclusion

Our study assessed fall risk in adults with SMA by examining gait speed and fatigability. A trend emerged indicating that lower gait speeds might increase an individual’s risk of falls. The data also suggested a more pronounced decline in gait speed over the course of the 6MWT among fallers, highlighting fatigability as a possible determinant of fall risk that requires further evaluation. Overall, the findings emphasize the importance of continuing to assess gait speed and fatigability in this population to better predict fall risk. Future research should not only continue to assess gait speed but also expand to include other gait parameters in real time using wearable devices.

## Data Availability

The original contributions presented in the study are included in the article/supplementary material, further inquiries can be directed to the corresponding author.
